# The Lake Chad Basin, an Isolated and Persistent Reservoir of *Vibrio cholerae* O1: A Genomic Insight into the Outbreak in Cameroon, 2010

**DOI:** 10.1371/journal.pone.0155691

**Published:** 2016-05-18

**Authors:** Rolf S. Kaas, Antoinette Ngandjio, Ariane Nzouankeu, Achiraya Siriphap, Marie-Christine Fonkoua, Frank M. Aarestrup, Rene S. Hendriksen

**Affiliations:** 1 National Food Institute, Technical University of Denmark, Research Group for Genomic Epidemiology, WHO Collaborating Center for Antimicrobial Resistance in Foodborne Pathogens and European Union Reference Laboratory for Antimicrobial Resistance, Kgs. Lyngby, Denmark; 2 Centre Pasteur du Cameroon, Service Hygiène et Environnement section Microbiologie, P.O. Box 1274, Yaoundé, Cameroon; 3 Centre Pasteur du Cameroon, Laboratory of Bacteriology, P.O. Box 1274, Yaoundé, Cameroon; 4 Department of Microbiology and Parasitology, Faculty of Medical Science, University of Phayao, Phayao, 56000, Thailand; 5 Department of Microbiology, Faculty of Public Health, Mahidol University, Bangkok, 10400, Thailand; Beijing Institute of Microbiology and Epidemiology, CHINA

## Abstract

The prevalence of reported cholera was relatively low around the Lake Chad basin until 1991. Since then, cholera outbreaks have been reported every couple of years. The objective of this study was to investigate the 2010/2011 *Vibrio cholerae* outbreak in Cameroon to gain insight into the genomic make-up of the *V*. *cholerae* strains responsible for the outbreak. Twenty-four strains were isolated and whole genome sequenced. Known virulence genes, resistance genes and integrating conjugative element (ICE) elements were identified and annotated. A global phylogeny (378 genomes) was inferred using a single nucleotide polymorphism (SNP) analysis. The Cameroon outbreak was found to be clonal and clustered distant from the other African strains. In addition, a subset of the strains contained a deletion that was found in the ICE element causing less resistance. These results suggest that *V*. *cholerae* is endemic in the Lake Chad basin and different from other African strains.

## Introduction

Cholera is a serious and potential life-threatening waterborne communicable disease caused by *Vibrio cholerae* [1,2]. The disease is associated with poor water quality and inadequate sanitation, [3] and is transmitted by the fecal-oral route [4,5]. Cholera outbreaks are commonly reported and often related to collateral damage of natural disasters or flooding [6–9]. However, asymptomatic healthy carriers have also been observed, mainly infected with the El Tor biotype [10].

*V*. *cholerae* produces the hallmark of cholera; the enterotoxin: CTXφ and is classified into approximately 200 serogroups of which O1 and O139 are mostly associated with clinical cases and have the potential to cause endemic cholera [11–14]. The serogroup O1 can be further subdivided into two biotypes; Classical and El Tor, but recently, variants of those biotypes have been reported [15,16]. In addition, each of the biotypes also display two distinct serotypes; Inaba and Ogawa [13].

Since 1817, when cholera spread from the Indian sub-continent, seven pandemics have been observed. In 1961, the seventh pandemic began in Southeast Asia caused by O1 El Tor [14,17–20]. Whole genome sequence (WGS) analysis has identified eight distinct phyletic lineages; the lineages have been named L1-L8. Lineages L1 and L3-L6 represent the former pandemics and L2 represents the current, seventh, El Tor pandemic. Lineages L7 and L8 are formed by unique isolates [19].

In 2013, a total of 129,064 cholera cases were reported to the World Health Organization, of which, 43% came from Africa. However, due to underreporting and insufficient surveillance data, the true global burden is estimated to be significantly higher, from 1.4 to 4.3 million cases, and 28,000 to 142,000 deaths per year (http://www.who.int/gho/epidemic_diseases/cholera/cases_text/en/).

In this study, it was of interest to determine the genetic relatedness of contemporary clinical O1 El Tor *V*. *cholerae* isolates originating from the 2010 outbreak in Cameroon by a phylogenetic analysis based on WGS. The study also includes an analysis of the occurrence of antimicrobial resistance genes and the mechanisms hereof. In addition, we want to elucidate if Lake Chad basin is an isolated and persistent reservoir of cholera by temporally comparing the surrounding countries’ outbreak data and spatially available genomic data in a global context.

## Methods

### Country outbreak data

The number of annual cholera cases per country was obtained from WHO (http://apps.who.int/globalatlas/dataQuery/). The database was queried for the total number of cases between 1970 and 2012, for each of the four countries surrounding Lake Chad (Cameroon, Chad, Niger, and Nigeria). The case numbers were imported into Excel and a stacked area plot was created (accumulated values).

### Samples and bacterial isolates

Twenty-four O1 El Tor *V*. *cholerae* isolates were obtained from stool samples collected from patients diagnosed with cholera between 2010 and 2011 in Cameroon.

### Antimicrobial susceptibility testing

The 24 O1 El Tor *V*. *cholerae* isolates were tested for antimicrobial susceptibility to ampicillin (AMP), azithromycin (AZI), cefotaxime (FOT), chloramphenicol (CHL), ciprofloxacin (CIP), gentamicin (GEN), meropenem (MERO), nalidixic acid (NAL), sulfamethoxazole (SMX), ceftazidime (TAZ), tetracycline (TET), tigecycline (TGC), and trimethoprim (TMP). The testing was performed by broth microdilution to determine minimum inhibitory concentration (MIC) with a commercially prepared panel of dehydrated antimicrobials (Sensititre; TREK Diagnostic Systems Ltd., East Grinstead, England). The MIC results were interpreted according to the Clinical and Laboratory Standards Institute (CLSI) breakpoints [21], except for tigecycline, for which the clinical breakpoint was used according to the recommendations of the European Committee on Antimicrobial Susceptibility Testing (EUCAST) (http://www.eucast.org). The *Escherichia coli* ATCC 25922 was used as reference strain for quality control, according to CLSI guidelines [21].

### Whole genome sequencing

Genomic DNA was extracted from the 24 O1 El Tor *V*. *cholerae* isolates using an Invitrogen Easy-DNA^TM^ Kit (Invitrogen, Carlsbad, CA, USA), and DNA concentrations were determined using the Qubit dsDNA BR assay kit (Invitrogen). The genomic DNA was prepared for Illumina using the Illumina (Illumina, Inc., San Diego, CA) NexteraXT® Guide 150319425031942 following the protocol revision C (http://support.illumina.com/downloads/nextera_xt_sample_preparation_guide_15031942.html). A sample of the pooled NexteraXT libraries was loaded onto an Illumina MiSeq reagent cartridge, using MiSeq Reagent Kit v2 and 500 cycles with a Standard Flow Cell. The libraries were sequenced using an Illumina MiSeq platform and MiSeq Control Software 2.3.0.3.

All 24 isolates were paired-end sequenced and ranged in insert size from 371 to 498 with an average of 427. The read coverage of the sequences was between 122X and 232X with an average of 187X.

Raw sequence data has been submitted to the European Nucleotide Archive (http://www.ebi.ac.uk/ena) under study accession no.: PRJEB13614 (http://www.ebi.ac.uk/ena/data/view/PRJEB13614). A complete list of genomic sequence data is available in the S1 Table.

The raw data was trimmed and cleaned for adapters using AdapterRemoval v. 1.1 (https://github.com/slindgreen/AdapterRemoval) before any analysis was performed.

VelvetK (http://bioinformatics.net.au/software.velvetk.shtml) was applied to each set of cleaned and trimmed data to estimate the k parameter for the following Velvet assembly, the k that provided a k-mer coverage closest to 20X.

VelvetOptimiser v. 2.2.5 (http://bioinformatics.net.au/software.velvetoptimiser.shtml) was used to test the range of the parameter k for each isolate and choose the optimal assembly. The range of k was set to the previously estimated k value +20 and -20 with a maximum of k = 99 and a minimum of k = 15. Velvet v. 1.2.07 [22] was used by VelvetOptimiser to do the actual *de novo* assemblies.

### Identification in silico of *V*. *cholerae*, antimicrobial resistance genes, SXT element, and the class 1 integron

The assembled sequences were analyzed to identify the species-specific gene (*ompW*), serogroup-specific genes (*rfbV*-O1, *wbfZ*-O139), the biotypes-specific genes (*ctxB*, *rstR*, *tcpA*), the MLST sequence type (ST) for *V*. *cholerae*, and the acquired antimicrobial resistance genes using the web-server MyDbFinder 1.0 with a selected threshold equal to 95% identity (https://cge.cbs.dtu.dk/services/MyDbFinder/) and the bioinformatic tool MLST (version 1.7) and ResFinder (version 2.1, 80% threshold for %ID/ 60% minimum length) available from Center for Genomic Epidemiology (CGE) [23,24].

The SXT element, the class 1 integron, and the presence of mutations in the DNA gyrase (*gyrA* gene) and in the DNA topoisomerase IV (*parC* gene) were determined using MyDbFinder. The nucleotide sequence of the integrase gene of the SXT element (*int*_*SXT*_ gene, AF099172) and the class 1 integron (*intI* gene, EU436855), and *gyrA*, and *parC* genes in the quinolone-resistant *V*. *cholerae* strains (GQ502315, KJ596550, and GQ502316) in GenBank were used as references.

### Phylogenetic structure of *V*. *cholerae* using single nucleotide polymorphisms

In order to put the genomic data from Cameroon in a global context, raw read data and assembled genomes from 352 *V*. *cholerae* strains were obtained from the European Nucleotide Archive (ENA) and GenBank, respectively. Only lineage 2 genomes belonging to the seventh El Tor pandemic were included in this investigation and only datasets that contained sufficient meta-data (collection date and location), however, there were a few exceptions (older strains missing location information).

Single Nucleotide Polymorphisms (SNPs) from the 24 O1 El Tor *V*. *cholerae* genomes from Cameroon as well as the 352 global *V*. *cholerae* strains were determined using the pipeline CSI Phylogeny 1.1 [25] available from the CGE website (https://cge.cbs.dtu.dk//services/all.php). Full genomic information is shown in S1 Table. Specific SNP information for the Cameroon strains is presented in S2 Table.

Briefly, the raw reads obtained from each isolate were mapped to the published completed reference strain N16961 (Acc. No.: NC_002505.1) using BWA version 0.7.2 [26]. SNPs were called using the ‘mpileup’ module in SAMTools version 0.1.18 [27]. Subsequently, SNPs were selected when meeting the following criteria: (i) a minimum distance of 10 bps between each SNP, (ii) a minimum of 10% of the average depth and at least 10X, (iii) a mapping quality greater than 30, (iv) a SNP quality greater than 25, (v) the position of the SNP was significantly covered in all the analysed isolates, (vi) in cases with contradicting calls, the called base must obtain a Z-score of at least 1.96 (corresponding to a p-value of 0.05), and (vii) all indels were excluded. The qualified SNPs from each genome were concatenated to a single pseudo-alignment.

For assembled genomes, Nucmer was used to align the contigs to a reference and call SNPs. The “show-snps” (with options “-CIlrT”) application was used to retrieve the SNPs. Both of these applications are part of the software package MUMmer v. 3.23 [28].

Maximum likelihood trees were created using FastTree [29]. FastTree was compiled with the accuracy alterations suggested by Aaron Darling (http://darlinglab.org/blog/2015/03/23/not-so-fast-fasttree.html). The non-lineage 2 strain M66-2 (Acc. No.: CP001233.1) was used to root the final tree.

## Results

### Epidemiological country data

**Fig 1:**
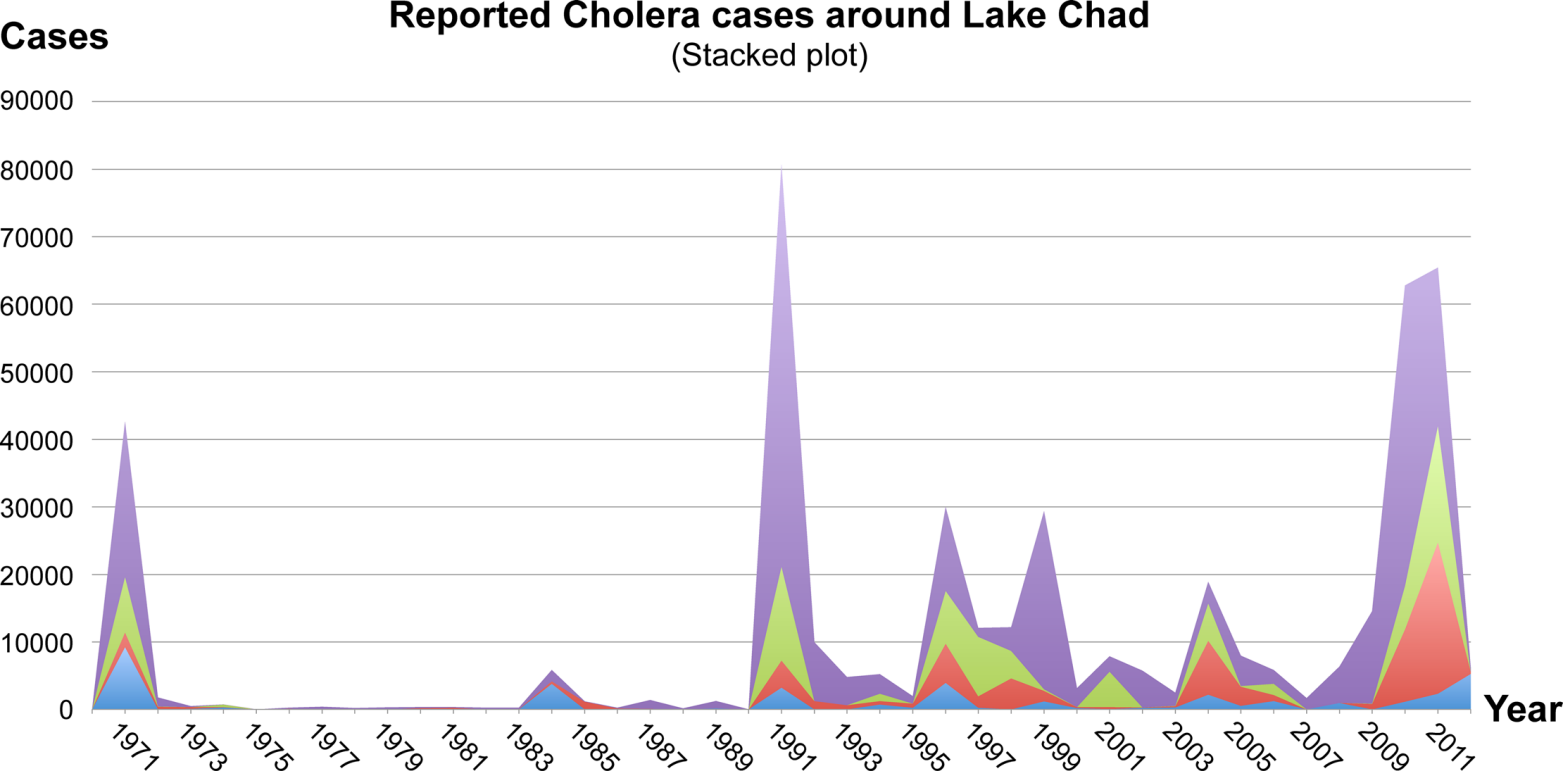
Reported cholera cases around the Lake Chad basin. The number of cases per year (1970–2012) presented in a cumulative graph. The following colors represent each country: Purple represents Nigeria, green represents Chad, red represents Cameroon, and blue represents Niger.

**Fig 2:**
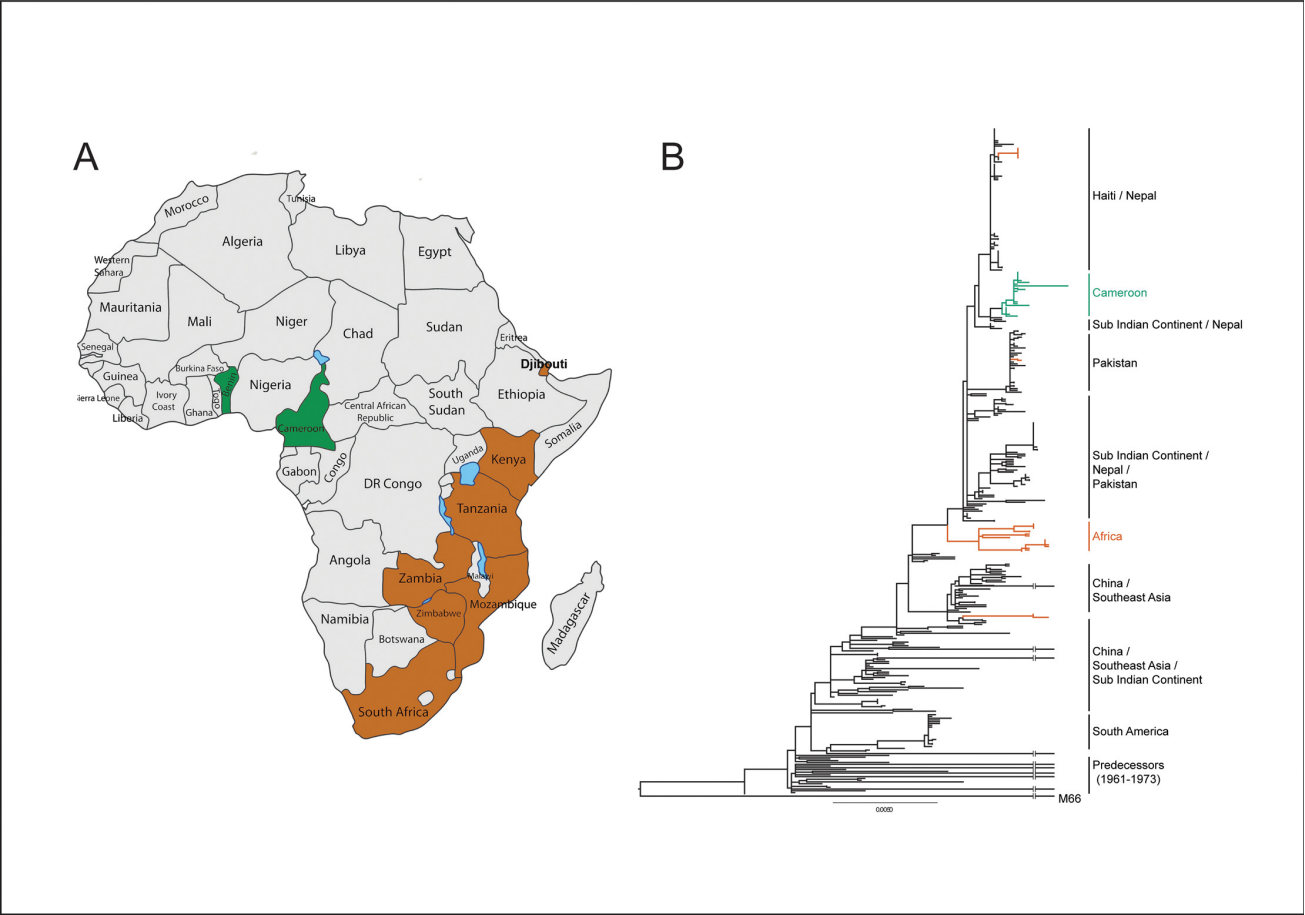
The inferred phylogeny based on SNPs for the Cameroon isolates. Each isolate is presented with a strain name followed by the city of origin and year of isolation. The vertical lines divide the isolates into two groups, representing the two different resistance profiles. The resistance genes that differ are marked in brown.

The emergence of the seventh cholera pandemic started around the Lake Chad basin in 1971 and led to more than 22,931, 9,265, and 8,230 cases in Nigeria, Niger, and Chad, respectively. In contrast, the 1971 outbreak affected Cameroon far less with only 2,167 cases http://www.who.int/cholera/countries/en/ (Fig 1). Between 1971 and 1991, only a few sporadic cases and minor outbreaks were reported from the Lake Chad basin, except for a single, larger isolated outbreak in Niger in 1984. A major outbreak among the four countries was observed in 1991; it was the largest outbreak ever recorded in Nigeria. Unfortunately, any information related to the cause seems to be unpublished. This outbreak coincides with the first emergence of atypical El Tor variants in the Ganges Delta area and could potentially be related due to the genomic clustering between genomes from Cameroon and the sub-Indian continent (Fig 2) [30]. Ever since, cholera has become endemic in the Lake Chad basin following a pattern of cross-border transmission among the four countries with large coinciding outbreaks reported from the area in the years 1996, 1998/1999, and 2004/2005/2006. In 2009, the latest coinciding outbreak in the region started and intensified in 2010/2011, affecting 41,787 people in Nigeria. In Cameroon and Chad, the outbreak likewise progressed with a total of 17,121 and 4,410 cases reported, respectively. In Niger, Chad, and Cameroon, the outbreak was the worst since 1971. The outbreak peaked in Nigeria (44,456 cases) in 2010, in Chad (17,267 cases) and Cameroon (22,433 cases) in 2011, and Niger (5,284 cases) in 2012 (Fig 1).

### Antimicrobial resistance, antimicrobial resistance genes, the class 1 integron and the SXT element

**Fig 3:**
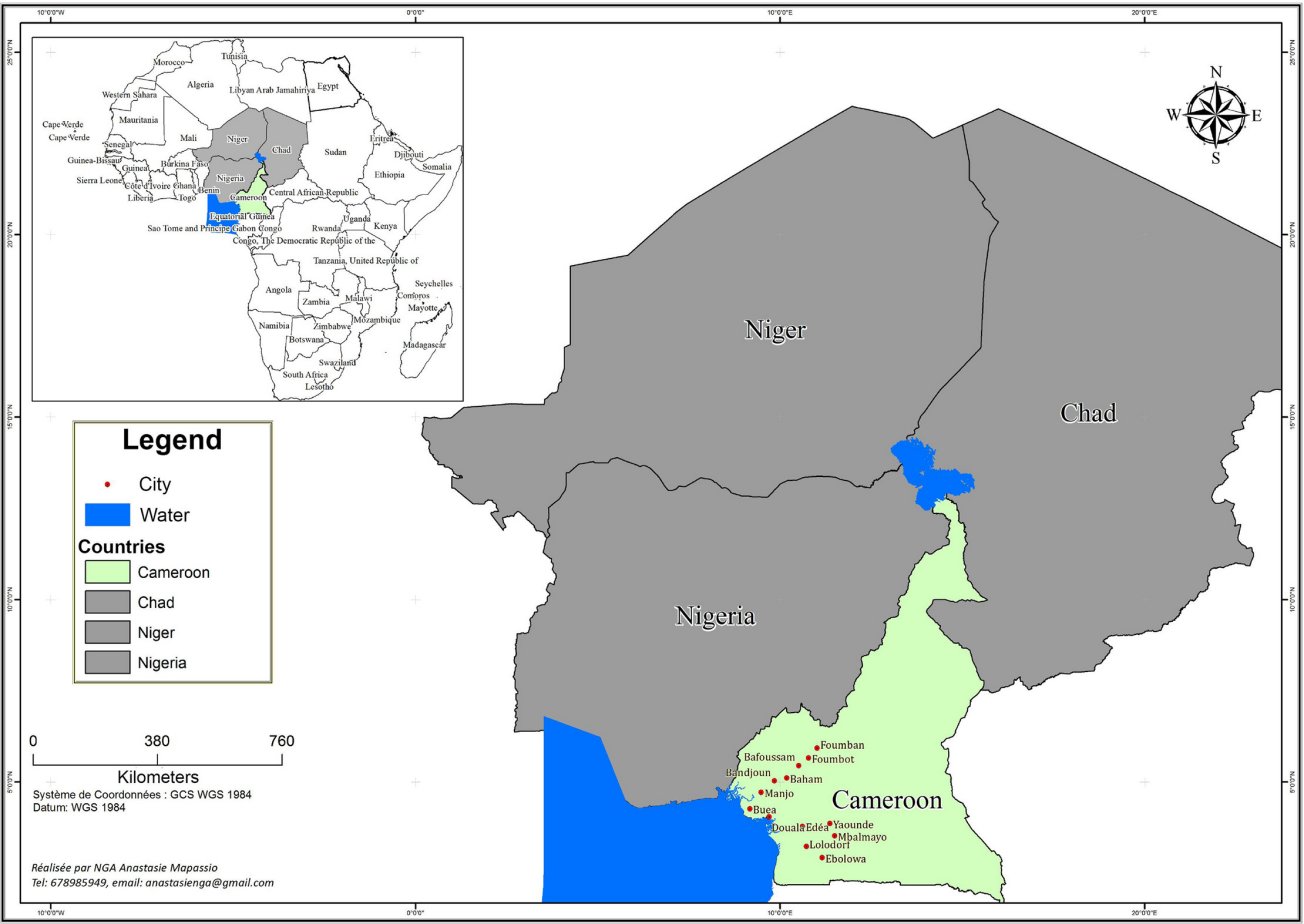
Location of *Vibrio cholerae* cases in Cameroon and around Lake Chad basin. Red dots in Cameroon indicate the location of the cities from where the isolates originate.

**Fig 4:**
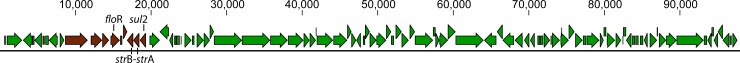
A graphical representation of the ICE fragment ICEVchInd5. The arrows represent the genes. The numbers indicate bp. position on the ICE fragment, with position 1 starting at the first bp of the fragment. The brown arrows represent the genes that were missing from the less-resistant sub-clone.

**Fig 5:**
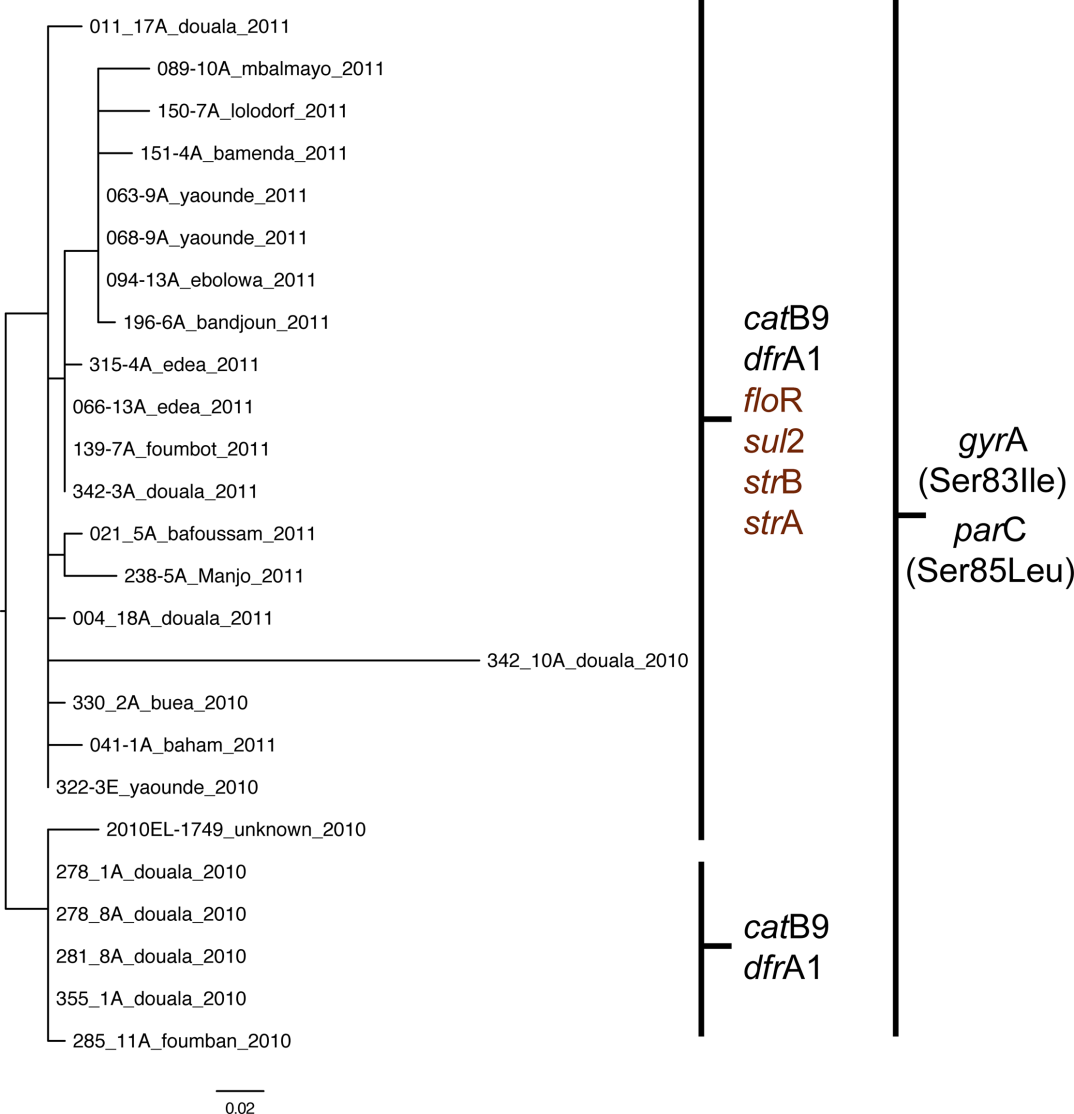
The Cameroon outbreak in a global perspective and phylogeny. Fig A. Map of Africa presenting the origin of the sequenced *V*. *cholerae* from the African continent. Green indicates Cameroon and Benin and brown indicates the rest of Africa. Fig B. Global phylogeny inferred from SNPs of 376 *V*. *cholerae* lineage 2 isolates. The origin of the majority of isolates in each cluster is indicated by the vertical lines to the right of the figure. The tree is rooted on the non-7^th^ pandemic strain M66-2. Ten branches have been shortened to fit the figure; this is indicated with a small gap flanked by vertical lines near the end of the branch.

The MIC determination and the corresponding antimicrobial resistance genes and amino acid substitutions of the 24 *V*. *cholerae* isolates revealed two different profiles with 19 isolates resistant to and harboring the following substitutions/genes: ciprofloxacin—two amino acid substitutions in *gyr*A (Ser83Ile) and *par*C (Ser85Leu); sulphonamides (*sul*2); streptomycin (*str*A/*str*B); florphenicol (*flo*R); chloramphenicol (*cat*B9); and trimethoprim (*dfr*A1); and five isolates only resistant to the latter two antimicrobials and corresponding genes (Fig 3). Of the five less-resistant isolates, four isolates (#278_1A, #278_8A, #281_8A, #355_1A) originated from Douala situated close to the sea, and one isolate (#285_11A) originated from Foumban, closest to Lake Chad (Fig 4). Interestingly, all isolates carried the integrating conjugative element (ICE) variant corresponding to ICEVchInd5 [31] with the notable difference that the five less-resistant isolates revealed a gap from approximately bp. position 8600 to 19400 in the ICE fragment excluding the four resistance genes; *sul*2, *str*A, *str*B, and *flo*R (Fig 5). The ICE variant was found using a new bioinformatic tool under development for typing of *V*. *cholerae* determined “VcTypeFinder”, which will be freely available online from the CGE website in 2016. The ICE variant result was confirmed with BLAST.

### Serogroup, serotype, biotype, MLST, population structure of *V*. *cholerae* based on SNPs and genomic elements

All 24 *V*. *cholerae* strains belonged to the O1 serogroup, the atypical El Tor biotype, the MLST type ST-69 and harbored the *ctx*B7 toxin (found *in silico*).

Overall, 61 different SNPs were found among the Cameroon strains with the genome #342_10A from Douala being substantially different from the others (Fig 3). The closest neighboring genome to #342_10A differed with 25 SNPs (#322_3E, Yaounde, 2010). Interestingly, the 25 SNPs were all singletons and only found in the genome #342_10A (S2 Table). The 25 SNPs, in general, does not seem to cluster, and from the reference genome annotation they do not seem to be located in mobile elements (S3 Table). The *mutS* gene was inspected to assess the presence of hypermutators but seemed functional. However, if the 25 SNPs were excluded from the analysis, #342_10A would be identical to #322_3E. In comparison, the greatest pairwise distance found between any of the other genomes were 17 SNPs. Thus, the outbreak isolates were all quite closely related and the phylogeny suggests the outbreak to be clonal. The less resistant sub-clones seemed to disappear in the beginning of the outbreak. However, it was not possible from the phylogenetic analysis to conclude whether the sub-clone was the original outbreak clone or a derivation of the more resistant sub-clone.

To establish a global perspective, the genomes from the Cameroon strains were compared with 352 *V*. *cholerae* genomes available from the European Nucleotide Archive (ENA) and GenBank, including African genomes originating from Kenya, Tanzania, Zambia, Malawi, Zimbabwe, Mozambique, South Africa, Djibouti, and Benin (Fig 2). The genomes from Cameroon and the one from Benin branched out from a clade consisting of genomes from the Sub-Indian continent and Nepal, whereas other genomes originating from Africa formed their own unique clade or were scattered among genomes of a global origin (Fig 2). Thus, confirming that the clone responsible for the Cameroon outbreak in 2010/2011 was an isolated clade in comparison with other African strains presented in the phylogenetic tree.

## Discussion

Not a lot of scientific typing data on cholera are available from the investigated region or in general from Africa. However, the outbreak around the Lake Chad basin in 2010 was previously believed to be caused by a multi-drug resistant atypical El Tor O1 *V*. *cholerae* strain similar to the Indian Orissa variant [32–35], which was confirmed in this study.

To date, only a few strains from the 2010 outbreak have been characterized by pulsed field gel electrophoresis (PFGE). The strains that have been characterized, generally show similar PFGE patterns and relationships to the ones from the 1971 outbreak [36]. In addition, only a single strain from Cameroon has been WGS in order to investigate possible links to the outbreak in Haiti in 2010 [37]. The true link between the reservoirs and countries requires a more advanced and appropriate genetic analysis such as a SNP analysis using WGS [38], as applied in this study to determine the clonality of the strains originating from the 2010/2011 outbreak in Cameroon. The topology of the global phylogenetic analysis performed in this study was in agreement with previous global studies of *V*. *cholera*e [19, 39]. This study showed that the clone responsible for the Cameroon outbreak in 2010/2011 was a single introduction and formed an isolated clade in comparison with other African strains presented in the global phylogenetic tree. This analysis combined with the epidemiological data from WHO support the hypothesis that *V*. *cholerae* is endemic to this area of Africa, and the Lake Chad basin is an isolated reservoir for cholera. To verify this hypothesis, isolates including also potentially stored historical strains from the other countries around Lake Chad should be obtained and sequenced. Unfortunately, the stability and laboratory capacity of these countries continues to be fragile, hampering sampling efforts.

There seems to be several major drivers responsible for the transmission of cholera and for it being endemic in the Lake Chad basin. Typically, the spread of cholera is caused by rain and flooding in endemic areas [32]. However, this seems not to be the case around the Lake Chad basin because several outbreaks have occurred in the dry seasons. However, several studies have reported outbreaks in areas of severe morbidity and mortality and caused by multiple factors such as poor sanitation, contaminated potable water, fecal-contaminated wells (due to run-off), travel, and trade [40–42]. This could potentially explain why minor clonal differences were observed in the genomes from Cameroon such as the loss of gene cassettes from the ICE fragment and how strains from different cities were related.

The antimicrobial susceptibility data and corresponding antimicrobial resistance indicated the presence of the SXT integrase harbored in the ICE. Previous studies from countries around the Lake Chad basin and of the same time period reported similar *V*. *cholerae* isolates resistant to trimethoprim (*dfr*A1), sulfonamides (*sul*2), nalidixic acid, and reduced susceptibility to ciprofloxacin caused by amino acid substitutions in the DNA gyrase, *gyrA* (Ser83Ile) and the DNA topoisomerase IV, *parC* (Ser85Leu) [34,36] which correspond well to the data of this study. In addition, resistance was also reported to chloramphenicol (*flo*R), ampicillin, and *str*A and *str*B in other studies [36,43]. These findings support the local phylogeny as well as the perception that cholera in the Lake Chad basin has been persistent and an isolated reservoir for decades.

The decline in WGS cost coupled with the growing availability of user-friendly free bioinformatic tools, such as the tools used in this study (CSI Phylogeny, MLST, ResFinder [23–25], and VcTypeFinder in development) enables researchers to do fast characterizations and globally link cholera outbreaks, enhancing the ability to control and understand *V*. *cholerae* in the future.

In conclusion, the Cameroon outbreak was found to be clonal with a single introduction. The genomes of the isolates were in general found to be very similar. However in the ICE, which was found to be of the ICEVchInd5 variant, a deletion was found in a small subset of the isolates; the deletion caused these isolates to be less resistant. The results presented in this study along with the case reports obtained from WHO supports the hypothesis that *V*. *cholerae* is endemic to the Lake Chad basin. It further suggests that it is clonal and different from other African *V*. *cholerae*, this however needs to be further investigated by inclusion of more and historical strains.

## Supporting Information

S1 Table Supplementary_table1-Sequence_info. (XLSX)

S2 Table Supplementary_table2-Cameroon_SNPs. (XLSX)

S3 Table Supplementary_table3-Cameroon_SNP_annotations. (XLSX)
